# List of reviewers 2021

**DOI:** 10.1192/bjo.2022.23

**Published:** 2022-03-07

On behalf of the editorial board, the Editor-in-Chief would like to thank the following for their contribution as peer reviewers in the period from January 2021 – December 2021. Their anonymous work for the journal forms the foundation to its success, and is highly appreciated.

- Melanie Abas
- Mohammed Abousaleh
- Balkozar Adams
- Leslie Adams
- Armita Adily
- Vivek Agarwal
- Bruno Agustini
- Suhana Ahmed
- Olesya Ajnakina
- Hasanen Al-Taiar
- Faruq Alam
- Regi Alexander
- Afia Ali
- Abdulmajeed Alkhamees
- Norah Almuhanna
- Daniel Alvarez-Fischer
- Flavia Alves
- Shereen Aly
- Ahmad Alzahrani
- Humma Andleeb
- Andrea de Angelis
- Afifa Anjum
- Paul Appelbaum
- S.M. Yasir Arafat
- Luis Araujo
- Dave Archard
- Angeliki Argyriou
- Kevin Ariyo
- Rehan Aziz
- Enrique Baca-Garcia
- Ajay Bakhla
- Ioannis Bakolis
- Azza Bakry
- David Baldwin
- Matteo Balestrieri
- Zoe Barczyk
- Kirsten Barnicot
- Lucie Bartova
- Markus Bassler
- Joy Noel Baumgartner
- Anna Baverstock
- Klaus Martin Beckmann
- Victoria Bein
- Victoria Bell
- Matthew Bennion
- Tomi Bergström
- Luca Bernardi
- Tom Berney
- Wade Berrettini
- Marco Bertelli
- Frank Besag
- Venkat Bhat
- Vishal Bhavsar
- Kamaldeep Bhui
- Andrew Bickle
- Eleonora Bielawska-Batorowicz
- Rebecca Bind
- Victoria Bird
- Catherine Bird
- Mengesha Biresaw
- Jonathan Bisson
- Asit Biswas
- Istvan Bitter
- Donald Black
- Rebecca Blackmore
- Jed Boardman
- Harm Boer
- Lana Bojanić
- Rohan Borschmann
- Hans Bosma
- Michel Botbol
- Theo Bouman
- Nick Bouras
- Juliette Bouzy
- Phillip Boyce
- Anders Boyd
- Tim Bradshaw
- Bethany Brand
- Erica Breuer
- Sofia Brissos
- Andrew Brittlebank
- Jillian Broadbear
- Carlinde Broeks
- Emma Broglia
- Konstantin Brückmann
- Xuan Bu
- Paola Bucci
- Dejan Budimirovic
- Oscar Bukstein
- Katherine Burdick
- Julian Burger
- Rochelle Burgess
- Wendy Burn
- Walter Busuttil
- Katherine Button
- Niels Buus
- Amanda Bye
- Eric Caine
- Elizabeth Camacho
- Jose Antonio Camacho Conde
- Andres Camilo Cardozo Alarcon
- Sara Carletto
- Marina Carpena
- Peter Carpenter
- Holly Carter
- Janet Carter
- Gustavo Carvalho de Oliveira
- Cecilia Casetta
- Patricia Casey
- Rakesh Chadda
- Samuel Chamberlain
- Carlyle Chan
- Tarani Chandola
- Prabha Chandra
- Imran Chaudhry
- Qingwei Chen
- Runsen Chen
- Gang Chen
- Andrew Cheng
- Helen Cheyne
- Rie Chiba
- Jennifer Chipps
- Lois Choi-Kain
- Ciaran S. Clarke
- Seonaid Cleare
- Catherine Clelland
- Caroline Clements
- Marylene Cloitre
- John Cookson
- Zafra Cooper
- Christine Cooper Vince
- Peter Cornwall
- Ana Costa
- Sara Costi
- Ken Courtenay
- Lesley Cousins
- Tom Craig
- John Craig
- Mike Crawford
- Matthew Croucher
- Marie Crowe
- Wiesław Cubała
- Ruth Cunningham
- Qin Dai
- Elias Dakwar
- Oliver Dale
- Christian Dalton-Locke
- Michael Daly
- Anna-Karin Danielsson
- Jayati Das-Munshi
- Oana David
- Thandi Davies
- Katrina Davis
- Enya Daynes
- Johannes De Kock
- Wendy Dean
- Kimberlie Dean
- Ian Deary
- Ana Teresa Delfino
- Kutay Demirkan
- Mekdes Demissie
- Brecht Devleesschauwer
- Annabella Digiorgio
- Gina Dimitropoulos
- Lora Dimitrova
- Jade Donaghy
- Martin Dorahy
- Katie Douglas
- Christopher Dowrick
- Małgorzata Dragan
- Bernadka Dubicka
- Coralie Dumoulin
- Julian Eaton
- Kate Eggleston
- Sharif El-Leithy
- Angelika Erhardt-Lehmann
- Javier Escobar
- Bruno Etain
- Kerri Evans
- Anne Eyre
- Hanan Ez elarab
- Nichole Fairbrother
- Hanna Falk Erhag
- Chao Fang
- Miguel Farias
- Lorna Farquharson
- Daniel Fatori
- Seena Fazel
- Abe Fekadu
- Johannes Fellinger
- Peter Fenwick
- Lucrezia Ferrante
- Laura Ferraro
- Emily Finch
- Mudasir Firdosi
- Claudia Fischer
- Emla Fitzsimons
- Lena Flyckt
- Marcella Fok
- Luciana Fonseca
- Leonardo Fontenelle
- Cheryl Foo
- Malcom Forbes
- Tamsin Ford
- David Foreman
- Andrew Forrester
- Sarah Forthal
- James Foulds
- Tanja Franciskovic
- Sophia Frangou
- Emma Fransson
- Mark Freestone
- Paul French
- Thomas Frodl
- Leiwen Fu
- Romayne Gadelrab
- Peter Gallagher
- Richard Gallagher
- Satheesh Kumar Gangadharan
- Caroline Gao
- Grace Gao
- Maria Gardani
- Elena Garralda
- Chiara Gastaldon
- Graham Gee
- Manoj George
- Galit Geulayov
- Nassir Ghaemi
- Cassandra Gheorghe
- Abhishek Ghosh
- Domenico Giacco
- Kristina Gicas
- Lucas Giner
- Michael Gitlin
- Paul Glue
- Brandon Goldstein
- Clara González Sanguino
- Sarah Gordon
- Christopher Gordon
- Louise Gorman
- Philippos Gourzis
- Linda Grabbe
- Denford Gudyanga
- Amelia Gulliver
- Mayank Gupta
- Kassahun Habtamu
- John Haltigan
- Johannes Hamann
- Elliot Hampsey
- Jens Peter Hansen
- Ketil Hanssen-Bauer
- Helen Harding
- Stephanie Hare
- Zinta Harrington
- Nigel Harvey
- Alex Haslam
- Tanya Hauck
- Aaron Hauptman
- Philip Hazell
- Claire Henderson
- Urs Hepp
- Catherine M. Herba
- Holly Herberman Mash
- Helen Herrman
- Morten Hesse
- Walter Hewer
- Madelyn Hsiao-Rei Hicks
- Christoph Hiemke
- Donald Hilty
- Lindsey Hines
- Yoshiyuki Hirano
- Anders Hjern
- Roger Ho
- Jennifer Hoblyn
- Nico Hoertel
- Maurício Hoffmann
- Josanne Holloway
- Frank Holloway
- Philipp Homan
- Ismail Hosen
- Sahadat Hossain
- Louise Howard
- Yong-Dong Hu
- Yueqin Huang
- Dieneke Hubbeling
- Peter Hughes
- Clara Humpston
- Richard Hunter
- Muhammad Husain
- Nusrat Husain
- Mark Huthwaite
- Christoph Hörmann
- Eduardo Iacoponi
- Maree Inder
- Felice Jacka
- Brian Jacobs
- Nicholas Jacobson
- Sneha Jadhav
- Peter James
- Imke Jansen
- Marek Jarema
- Arash Javanbakht
- Manish Jha
- Sheri Johnson
- David Jolley
- Edgar Jones
- Brett Jones
- Mark Jordans
- Martin Jorgensen
- Marin Jukic
- Mario Juruena
- Thomas Kabir
- Michael Kaess
- Ashraf Kagee
- Richard Kanaan
- Stephanie Kandsperger
- Hitoshi Kaneko
- Tyler Kaster
- Cornelius Katona
- Tamar Katz
- Kenneth Kaufman
- Harneet Kaur
- Matthew Kemptom
- Michael Kerr
- Jess Kerr-Gaffney
- Helen Killaspy
- Irina Kinchin
- Patricia Kinser
- Tayyeba Kiran
- James Kirkbride
- Steve Kisely
- Taro Kishi
- Stefan Kloiber
- Günter Klug
- Anke Kobach
- Yasin Koc
- Roman A Koposov
- Giorgio Kotzalidis
- Golo Kronenberg
- Krzysztof Krysta
- Ashok Kumar-Jainer
- Veena Kumari
- Andre Kuntz
- Hiroshi Kunugi
- Wiliiam LaFrance
- Danielle Lamb
- Matthew Large
- Celine Larkin
- Rachel Latham
- Sharon Lawn
- Stephen Lawrie
- Edouard Leaune
- Daniel Leightley
- Belinda Lennox
- Eric Lenze
- Peter Lepping
- Glyn Lewis
- Roberto Lewis-Fernandez
- Zezhi Li
- Ming Li
- Rodrigo Lima
- Jennie Lister
- Kathy Liu
- Xufeng Liu
- Shan-Yu Liu
- Adrian Lloyd
- Carmen Lopez
- Pablo Lopez
- Bernd Lowe
- Bartosz Łoza
- Peter Lucassen
- Mario Luciano
- Cecilia Lundin
- Tom Lundin
- Angela Lupattelli
- Andrew Lustig
- Jurjen Luykx
- Sean Lynch
- Zaza Lyons
- Deirdre MacManus
- Mariana Maciel
- Ambra Macis
- Nicola Magnavita
- Kathryn Magruder
- Rohan Mahabaleshwarkar
- Rajneesh Mahajan
- Jennifer Manuel
- Chen Mao
- Suzanne Mares
- Joanna Martin
- Denise Martin
- Allison Marziliano
- Joanna Maselko
- Jolanta Masiak
- Lorenzo Mazzarini
- Rosalind McAlpine
- Jane McClinchy
- David McDaid
- Lauren McGillivray
- Andrew McKechanie
- Sally McManus
- Roisin McNaney
- Fiona Mcnicholas
- Alan Meehan
- Werkua Mekonnen
- Habtamu Mekonnen
- João Campos Mendes
- Matthew Menear
- Sarah Meshberg-Cohen
- Ricarda Mewes
- Christian Michael
- Georgina Miguel Esponda
- Ryan Mills
- Supriya Misra
- Francis Mitrou
- Fhionna Moore
- R. O. Moreira
- Emma Morton
- Christoph Mueller
- Marco Mula
- David Mumford
- Trine Munk-Olsen
- Esther Murray
- Katherine Musliner
- Matthias Müller
- Dieter Naber
- Jhunu Nahar
- Miharu Nakanishi
- Vamanjore Naushad
- Laura Nawijn
- David Ndetei
- Diana Nechita
- Katlyn Nemani
- Minh-Hoang Nguyen
- Neil Nixon
- Evangelos Ntontis
- Peter Nussbaum
- Maria Nystazaki
- David O' Regan
- Aileen O'Brien
- Joanne O'Connor
- Rory O'Connor
- Lydia O'Sullivan
- Yuko Odagiri
- Sherifat Oduola
- Tadahisa Ohara
- Tamara Ondruskova
- Sian Oram
- Abigail Ortiz
- Mugtaba Osman
- Edoardo Ostinelli
- Dennis Ougrin
- Simple Ouma
- Clare Pain
- Lawrence Palinkas
- Fryni Panayidou
- Daniel Pankowski
- Carmine Pariante
- Gordon Parker
- Glenys Parry
- Andrew Peckham
- Julia Pei
- Jonathan Peirce
- Eva Petkova
- Theodore Petti
- Peter Phiri
- Giulia Piazza
- Alex Pieterse
- Toby Pillinger
- Vanessa Pinfold
- Alexandra Pitman
- Michael Pluess
- Rob Poole
- Richard Porter
- Maj-Britt Rocio Posserud
- Iskra Potgieter
- Andrew Powell
- Annabel Price
- Stefan Priebe
- Eleonora Prina
- Soorej Jose Puthoopparambil
- Yann Quide
- Samah Rabei
- Ravi Ramasamy
- Rosalind Ramsay
- Ambiga Ravi
- Ursula Read
- Sara Real Castelao
- Cheryl Rees
- Susan Rees
- Sarah Reeve
- Cheryl Regehr
- Abraham Reichenberg
- Lennart Reifels
- David Reiss
- Gaby Resmark
- Charles Reynolds, III
- Ali Rezaeisharif
- Abdul Rhouma
- Wagner Ribeiro
- Simon Rice
- Laura Ridgeway
- Zoltan Rihmer
- Louise Robinson
- Jo Robinson
- Tomos Robinson
- Paul Robinson
- Humbelina Robles Ortega
- Deborah Robson
- Liana Romaniuk
- Stuart Rosen
- Larissa Rossen
- Ashok Roy
- Paola Rucci
- Antonio Ruiz Garcia
- Augustus Rush
- Torleif Ruud
- Grace Ryan
- Janus Rybakowski
- Martha Sajatovic
- Estela Salagre
- Myrto Samara
- Musa Sami
- Rahil Sanatinia
- María Dosil Santamaría
- Vedat Sar
- Amir Sariaslan
- Alan Schatzberg
- Diana Schendel
- Stephen Schoenthaler
- Jan Scott
- Edward Selby
- Lucas Sempe
- Vaheshta Sethna
- Nick Sevdalis
- Arieh Shalev
- Rohit Shankar
- Jalil-Ahmad Sharif
- Jenny Shaw
- James Shearer
- Bryony Sheaves
- Bart Sheehan
- Lin Shen
- Andrew Shepherd
- Sukhi Shergill
- Mark Shevlin
- Rahul Shidhaye
- Sonali Shinde Tesia
- Brian Shiner
- Wendy Shoesmith
- Gemma Sicouri
- Derrick Silove
- Benedetta Silva
- Douglas Simkiss
- Judit Simon
- Alexander Simpson
- Carra Simpson
- Mark Sinyor
- James Smith
- Andrew Sommerlad
- Anne Sonley
- Faye Stanage
- John Stancombe
- Corbin Standley
- Thomas Steare
- Lucy Stephenson
- Robert Stewart
- Anne Stiggelbout
- Julia Stingl
- John Stone
- Rebecca Strawbridge
- Sladjana Štrkalj Ivezić
- Genichi Sugihara
- Paul Summergrad
- Lois Surgenor
- Nursel Sürmelioğlu
- Keith Syrett
- Olalla Sáiz-Vazquez
- Somayyeh Taklavi
- Sandila Tanveer
- Alvin Kuowei Tay
- Neil Thomas
- Lindsay Thomson
- Gillian Thomson
- Paul Tiffin
- Ami Tint
- Elena Toffol
- Elena Tomba
- Leonardo Tondo
- Ivan Torres
- Ellen Townsend
- Derek Tracy
- Samuel Tromans
- Hector Tsang
- Dimosthenis Tsapekos
- Mekonnen Tsehay
- Rebecca Turner
- Bryan Tysinger
- Michael Udedi
- Rachel Upthegrove
- Jolien van Breen
- Rob van den Brink
- Peter van der Velden
- Patrizia Velotti
- Pieter Ventevogel
- Lucia Verginer
- Simone Vigod
- Birgit Vollm
- Louiza Voniati
- Robert Walker
- Sonya Wallbank
- Malin Wallin
- Angel Wang
- Wayne Warburton
- Nicola Warren
- Danuta Wasserman
- Lauren Waterman
- Marielle Wathelet
- Rebecca Webb
- Myrna Weissman
- Karen Wetherall
- Daniel Whiting
- Richard Whittington
- Alina Wilkowska
- Jill Williams
- Richard Williams
- Chris Williams
- Claire Wilson
- Ceri Wilson
- Robert Winterhalder
- Michael Wise
- Ben Hoi-Ching Wong
- Amanda Woodrow
- Adam Włodarczyk
- Xiaoxian Xie
- Min Yang
- Xinhua Yang
- Lakshmi N. Yatham
- Yung-Chieh Yen
- Dong Keon Yon
- Roseline Yong
- Allan Young
- Yu Yu
- Sedigheh Zabihi
- Zainab Zadeh
- Roland Zahn
- Caroline Zangani
- Daniel Zheng
- Chuanjun Zhuo
- Hannah Ziobrowski

